# Supplementary material for: Comparative genomics provides new insights into the diversity, physiology, and sexuality of the only industrially exploited tremellomycete: Phaffia rhodozyma
Source: BMC Genomics. 2016 Nov 9;17:901. doi: 10.1186/s12864-016-3244-7 (PMC5103461; doi:10.1186/s12864-016-3244-7)
Supplement: Additional file 6: — List of orphan genes with links to PFAM (related to Additional file 1: Table S1). (ZIP 1428 kb) [file 12864_2016_3244_MOESM6_ESM.zip › PFAM_UNIQUE/G04771_P.html]

#                                                                            --- full sequence --- -------------- this domain -------------   hmm coord   ali coord   env coord
  
# target name        accession   tlen query name           accession   qlen   E-value  score  bias   #  of  c-Evalue  i-Evalue  score  bias  from    to  from    to  from    to  acc description of target
  
#------------------- ---------- ----- -------------------- ---------- ----- --------- ------ ----- --- --- --------- --------- ------ ----- ----- ----- ----- ----- ----- ----- ---- ---------------------
  
G04771\_P             -             93 Bd3614\_N             PF14442.1    138     0.011   14.4   0.5   1   1   1.2e-05     0.014   14.0   0.5    87   116    47    76    36    89 0.75 - Bd3614-like deaminase N-terminal  
G04771\_P             -             93 CENP-B\_dimeris       PF09026.5    101      0.57    9.1   4.6   1   1    0.0046       0.8    8.7   4.6    10    42    58    79    49    89 0.55 - Centromere protein B dimerisation domain  
G04771\_P             -             93 Cyclin               PF08613.6    149     0.011   14.8   0.0   1   1   3.1e-05     0.014   14.5   0.0   112   148     1    37     1    38 0.95 - Cyclin  
G04771\_P             -             93 DUF4611              PF15387.1     96     0.028   13.2   0.3   1   1   0.00012     0.031   13.1   0.3    59    80    53    76    22    85 0.71 - Domain of unknown function (DUF4611)  
G04771\_P             -             93 Nucleoplasmin        PF03066.10   149     0.071   11.3   0.4   1   1   0.00088      0.08   11.1   0.4   118   137    58    77    16    82 0.59 - Nucleoplasmin  
G04771\_P             -             93 PBP1\_TM              PF14812.1     81       1.1    8.3   4.5   1   1    0.0096       1.5    7.8   4.5    34    51    60    77    45    81 0.43 - Transmembrane domain of transglycosylase PBP1 at N-terminal
